# *Campylobacter jejuni *induces transcellular translocation of commensal bacteria via lipid rafts

**DOI:** 10.1186/1757-4749-1-2

**Published:** 2009-02-03

**Authors:** Lisa D Kalischuk, G Douglas Inglis, Andre G Buret

**Affiliations:** 1 Department of Biological Sciences, Inflammation Research Network, University of Calgary, Calgary, AB, Canada; 2 Agriculture and Agri-Food Canada, Lethbridge, AB, Canada

## Abstract

**Background:**

*Campylobacter *enteritis represents a risk factor for the development of inflammatory bowel disease (IBD) via unknown mechanisms. As IBD patients exhibit inflammatory responses to their commensal intestinal microflora, factors that induce translocation of commensal bacteria across the intestinal epithelium may contribute to IBD pathogenesis. This study sought to determine whether *Campylobacter *induces translocation of non-invasive intestinal bacteria, and characterize underlying mechanisms.

**Methods:**

Mice were infected with *C. jejuni *and translocation of intestinal bacteria was assessed by quantitative bacterial culture of mesenteric lymph nodes (MLNs), liver, and spleen. To examine mechanisms of *Campylobacter*-induced bacterial translocation, transwell-grown T84 monolayers were inoculated with non-invasive *Escherichia coli *HB101 ± wild-type *Campylobacter *or invasion-defective mutants, and bacterial internalization and translocation were measured. Epithelial permeability was assessed by measuring flux of a 3 kDa dextran probe. The role of lipid rafts was assessed by cholesterol depletion and caveolin co-localization.

**Results:**

*C. jejuni *81–176 induced translocation of commensal intestinal bacteria to the MLNs, liver, and spleen of infected mice. In T84 monolayers, *Campylobacter*-induced internalization and translocation of *E. coli *occurred via a transcellular pathway, without increasing epithelial permeability, and was blocked by depletion of epithelial plasma membrane cholesterol. Invasion-defective mutants and *Campylobacter*-conditioned cell culture medium also induced *E. coli *translocation, indicating that *C. jejuni *does not directly 'shuttle' bacteria into enterocytes. In *C. jejuni*-treated monolayers, translocating *E. coli *associated with lipid rafts, and this phenomenon was blocked by cholesterol depletion.

**Conclusion:**

*Campylobacter*, regardless of its own invasiveness, promotes the translocation of non-invasive bacteria across the intestinal epithelium via a lipid raft-mediated transcellular process.

## Background

Patients with IBD appear to display aberrant inflammatory responses to their commensal intestinal microflora via unknown mechanisms [1]. Normally, the intestinal microflora is effectively confined to the lumen by the epithelium. However, intestinal epithelial barrier defects may contribute to the development of IBD, as bacteria that translocate through the epithelium may expose submucosal immune cells to inappropriate antigenic stimulation and incite an inflammatory response towards the commensal microflora [2]. In combination with genetic and environmental factors, acute bacterial infection may initiate or exacerbate inflammation in IBD patients [3-8]. While specific mechanisms involved remain unknown, it is thought that the intestinal injury incurred during enteritis may facilitate translocation of luminal antigens [7]. In susceptible individuals, inflammation may become self sustaining due to ineffective down-regulation, despite elimination of the pathogen.

Intestinal bacteria can gain access to the lamina propria via a paracellular route, in which bacteria translocate between disrupted epithelial tight junctions ('leaky gut') [2]. For example, there is a correlation between increased intestinal paracellular permeability and bacterial translocation [9], and bacteria have been observed within the paracellular space of polarized enterocyte monolayers [10]. Bacteria may also translocate across the intestinal epithelium via a transcellular route, involving endocytic uptake followed by intracellular trafficking. For example, commensal intestinal bacteria have been observed within the cytoplasm of enterocytes in patients with IBD, via mechanisms that remain obscure [11]. As well, translocation of intestinal microflora can occur despite normal intestinal paracellular permeability [12]. Furthermore, translocation of non-invasive *E. coli *in enterocytes treated with the proinflammatory cytokine, interferon gamma (IFN-γ), occurs via a transcellular mechanism that precedes disruption in tight junction integrity [13].

*Campylobacter *species, including *C. jejuni*, *C. coli*, and *C. fetus*, are one of the most prevalent causes of human acute bacterial enteritis ('campylobacteriosis') in the developed world [14]. Disease is typically self-limiting and characterized by fever, abdominal pain, and inflammatory diarrhea [14]. Campylobacteriosis is the commonest risk factor for post-infectious irritable bowel syndrome, which occurs in ≈20–30% patients following infection [15]. Moreover, for reasons that are poorly understood, there is an increased risk of developing IBD in the first year following campylobacteriosis [4].

*C. jejuni *has been shown to disrupt the integrity of the intestinal barrier by targeting epithelial tight junctions [16,17]; however, whether this promotes translocation of non-invasive luminal bacteria is unknown. Using complementary models *in vivo *and *in vitro*, the objectives of this study were: (1) to determine whether *Campylobacter *induces translocation of non-invasive bacteria; and (2) to characterize underlying mechanisms. Results indicate that *Campylobacter *may induce translocation of non-invasive intestinal bacteria via a lipid raft-mediated transcellular process.

## Results

### *C. jejuni *induces bacterial translocation of commensal bacteria *in vivo*

Large numbers (*P *< 0.01) of microaerobic bacteria were isolated from the MLNs, liver, and spleen of *C. jejuni*-treated mice (3.08 ± 0.46, 2.03 ± 0.77, 3.12 ± 0.69 log_10 _CFU/g, respectively), compared to control mice (0, 0, 0.43 ± 0.43 log_10 _CFU/g, respectively). Bacteria were identified as *Proteus*, *Acinetobacter*, and *Pseudomonas*. *C. jejuni *were also isolated from the MLNs, liver, and spleen of all *C. jejuni*-treated mice (8/8) but not from uninfected controls (*P *< 0.001). Amp^R ^*E. coli *were isolated from the MLNs of 3/8 and the spleen of 1/8 *C. jejuni*-treated mice, but were not isolated from the liver of *C. jejuni*-treated mice, nor the MLNs, liver, or spleen of control mice.

### *C. jejuni *induces translocation of non-invasive *E. coli *across confluent epithelia

**Figure 1:**
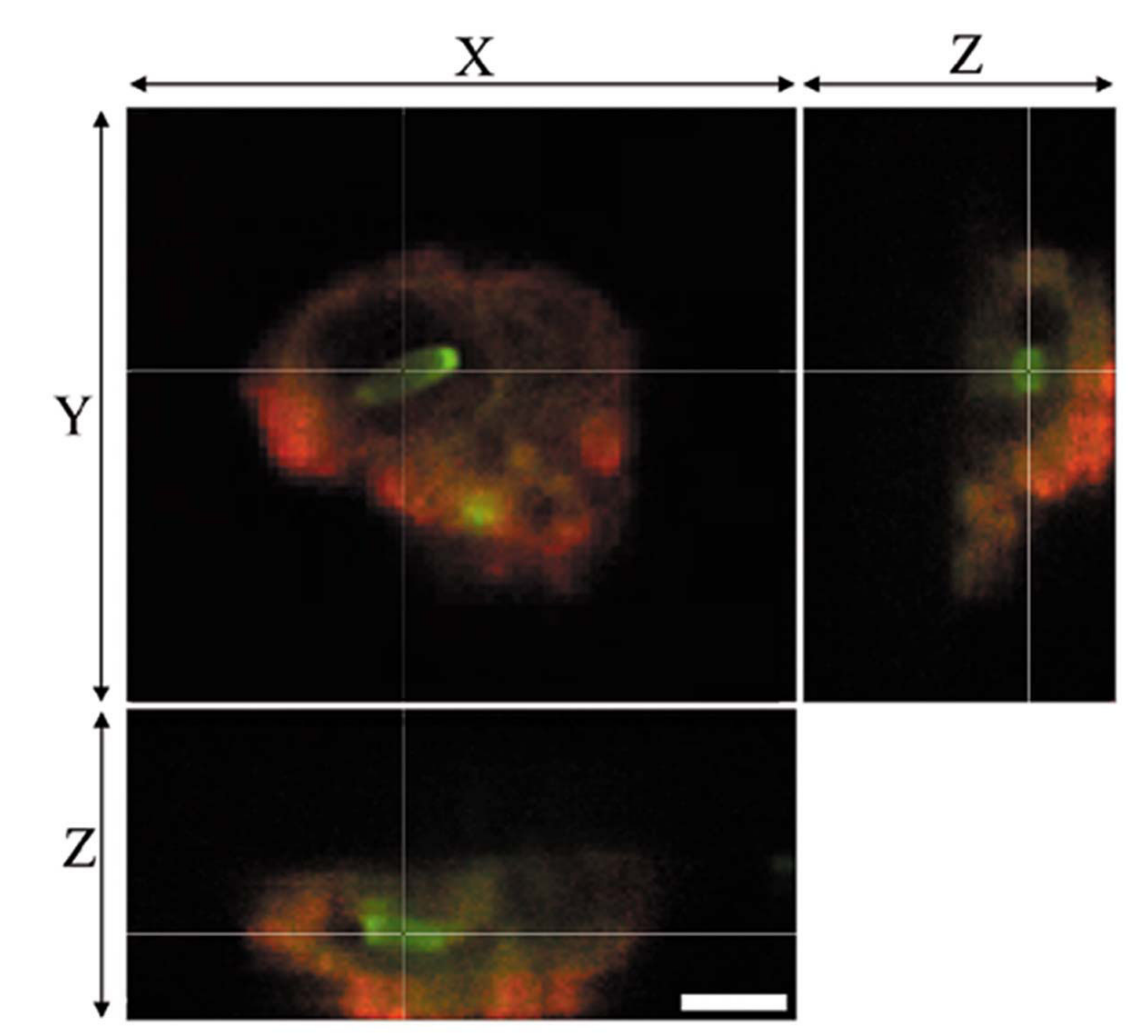
**Intracellular *E. coli *within *Campylobacter*-treated enterocytes**. Non-invasive *E. coli *HB101 (green) is localized in a membrane-bound vacuole (red) within *C. jejuni*-treated T84 enterocytes. Note confocal sectioning (i.e., x-z and y-z planes) showing cytoplasmic localization of the *E. coli*. Bar, 3 μM.

*E. coli *translocation was increased (*P *= 0.03) in *C. jejuni*-treated monolayers (2.41 ± 0.23 log_10 _CFU/ml), compared to controls (1.34 ± 0.17 log_10 _CFU/ml). *E. coli *internalization was also increased (*P *= 0.04) in *C. jejuni*-infected monolayers (2.09 ± 0.15 log_10 _CFU/ml), compared to controls (2.83 ± 0.04 log_10 _CFU/ml). Permeability was not different (*P *= 0.75) between treatments (0.035 ± 0.011% versus 0.031 ± 0.009% apical FITC-dextran/cm^2^/h for control and *C. jejuni*-treated monolayers, respectively). *E. coli *were visualized within membrane-bound vacuoles within the cytoplasm of *C. jejuni*-treated monolayers (Figure 1), but were not observed in controls (not shown).

### *C. jejuni*-induced translocation of *E. coli *is cholesterol-dependent

Treatment with MβCD or soluble cholesterol effectively decreased (*P *< 0.001) or increased (*P *< 0.001) the cholesterol of content of monolayers (9.68 ± 0.59, 1.78 ± 0.23, 24.18 ± 1.73 μg cholesterol/mg protein for untreated, MβCD-treated, and cholesterol-treated monolayers, respectively).

**Figure 2:**
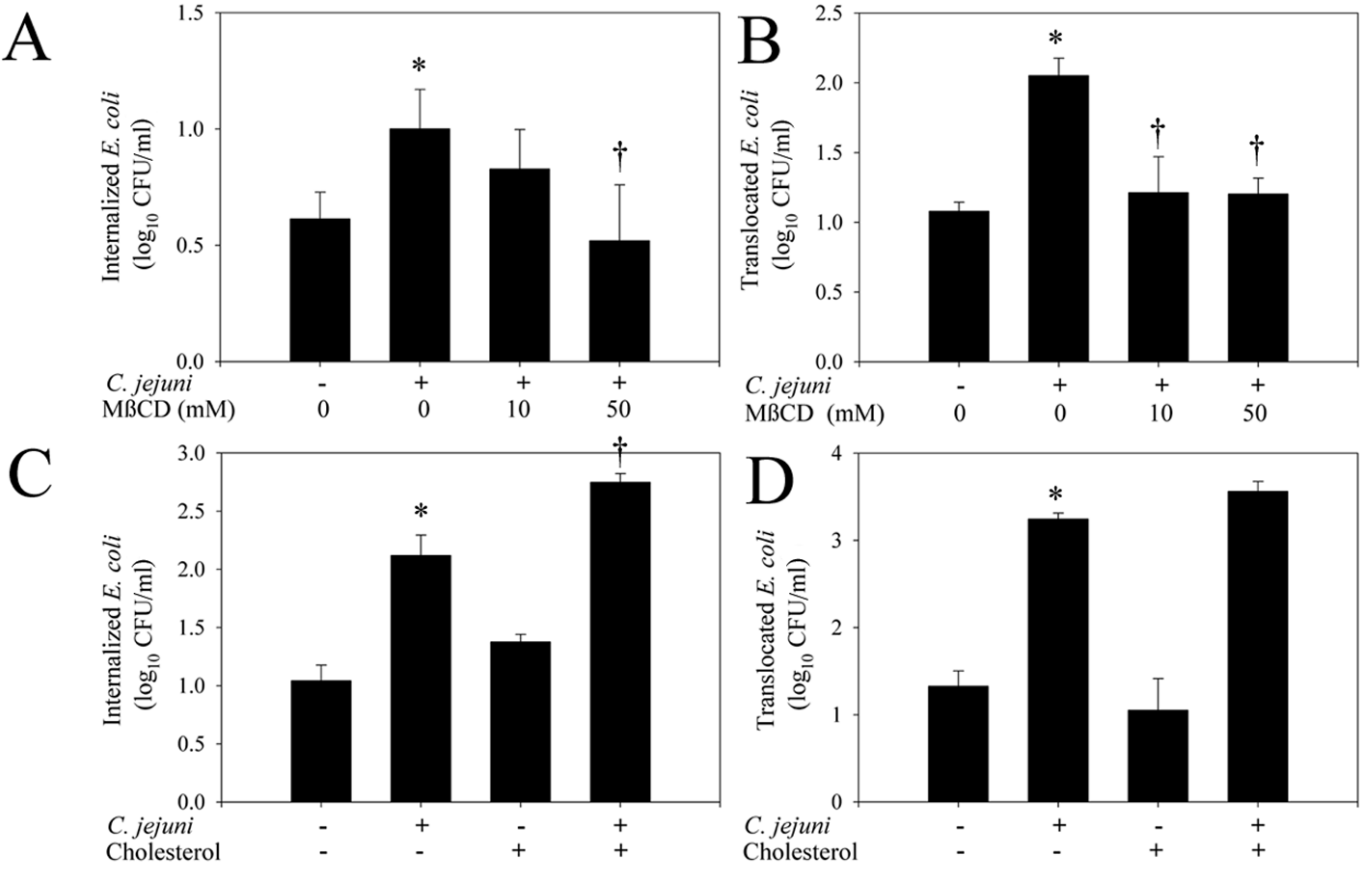
**Plasma membrane cholesterol affects *C. jejuni*-induced *E. coli *internalization and translocation**. Cholesterol depletion prevents *C. jejuni *81–176-induced (A) internalization, and (B) translocation of *E. coli *HB101 in confluent T84 monolayers, 4 h post-infection. (n = 3). Increased plasma membrane cholesterol promotes *C. jejuni *81–176-induced internalization (C) of *E. coli*, but does not affect *E. coli *translocation (D), 4 h post-infection. (n = 4). Data are means ± SEM, *P < 0.05 compared to untreated non-infected monolayers. †*P *< 0.05 compared to untreated *C. jejuni*-infected monolayers.

As observed previously, infection with *C. jejuni *significantly increased *E. coli *internalization (*P *= 0.03). In *C. jejuni*-treated monolayers, treatment with the lipid raft disruptor MβCD inhibited *E. coli *internalization (*P *= 0.04) and translocation (*P *= 0.04; Figure 2A and 2B). Internalization and translocation of *E. coli *were not different (*P *= 0.60 and *P *= 0.64, respectively), between *C. jejuni*/MβCD-treated and untreated monolayers. In *C. jejuni*-treated monolayers, there was increased internalization (*P *= 0.03) but not translocation (*P *= 0.08) of *E. coli *in monolayers that were treated with soluble cholesterol to augment plasma membrane cholesterol (Figure 2C and 2D). Internalization of *E. coli *in untreated and uninfected-cholesterol-treated monolayers was not different (*P *= 0.07). Cholesterol depletion and augmentation did not affect the invasion or translocation *C. jejuni *(results not shown).

**Figure 3:**
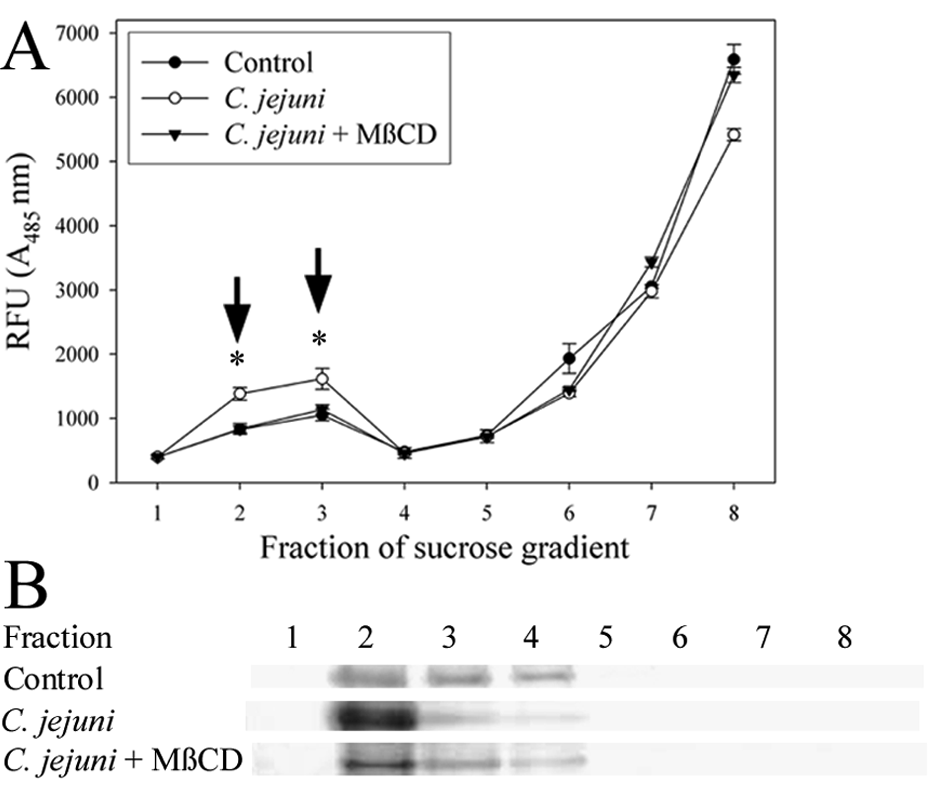
***C. jejuni *promotes association of *E. coli *with lipid rafts**. (A) Lipid rafts were isolated by sucrose gradient fractionation and fluorescent *E. coli *in each fraction was measured (values are shown as relative fluorescence units (RFU)). Arrows indicate fractions containing lipid raft-associated *E. coli*. *P < 0.05 compared to control and cholesterol-depleted monolayers. Data are means ± SEM (n = 3). (B) Fractions were analyzed by western blot analysis for the lipid raft marker, caveolin-1. A representative image from three experiments is shown.

Lipid rafts were isolated based on their buoyancy, by sucrose gradient fractionation (Figure 3), and were present in fractions 2 and 3 (as indicated by the presence of the lipid raft marker, caveolin-1). Fluorescent *E. coli *detected in fractions 2 and 3 represent lipid raft-associated *E. coli*, whereas the fluorescence in fractions 6–8 represents extracellular *E. coli*. Infection with *C. jejuni *caused *E. coli *to associate with lipid rafts. This effect was abolished by cholesterol depletion with MβCD.

**Figure 4:**
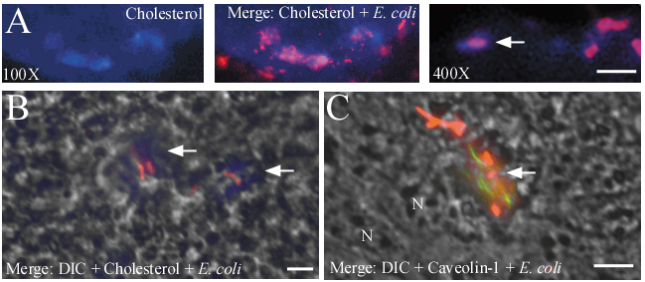
***C. jejuni *81–176 causes accumulation of cholesterol and promotes co-localization of *E. coli *HB101 with lipid rafts**. Epifluorescent images demonstrating (A) accumulation of cholesterol (blue), and co-localization of *E. coli *(orange-red) with (A, B) cholesterol and (C) caveolin-1 (green), 4 h post-infection. DIC, differential interference contrast. N, nucleus. Arrows indicate co-localization of *E. coli *with lipid raft markers. Bar = 2 μM. Representative images are shown from two experiments performed in quadruplicate.

Epifluorescent microscopy was used to visualize the association of *E. coli *with cholesterol and caveolin-1 (Figure 4). Accumulations of cholesterol were observed in *C. jejuni*-treated monolayers (Figure 4A), wherein *E. coli *were co-localized (Figure 4A, B). *E coli *co-localized with caveolin-1 in *C. jejuni*-treated monolayers (Figure 4C). Accumulation of cholesterol and co-localization of *E. coli *with cholesterol or caveolin-1 were not observed in control monolayers (not shown).

### *E. coli *translocation does not correspond with *Campylobacter *invasiveness

**Figure 5:**
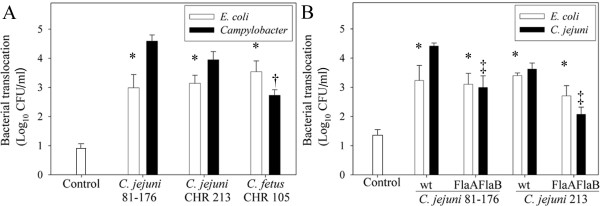
**Translocation of *E. coli *does not correspond with *Campylobacter *invasiveness**. (A) Translocation of *E. coli *HB101 (white bars) and *Campylobacter *(black bars) in T84 monolayers infected with highly (strain 81–176), moderately (CHR213), and modestly-invasive (CHR105) *Campylobacter *strains, 4 h post-infection. (B) Translocation of *E. coli *HB101 (white bars) and *Campylobacter *(black bars) in T84 monolayers treated with wild-type *C. jejuni *81–176 and CHR213, and corresponding invasion-defective FlaAFlaB mutants Data are means ± SEM (n = 3). *P < 0.05 compared to control monolayers. †*P *< 0.05 compared to *C. jejuni *81–176 and *C. jejuni *CHR213. ‡ P < 0.05 compared corresponding wild-type *C. jejuni*-treated monolayers.

*C. jejuni *strains with various degrees of invasiveness were used to assess whether *E. coli *translocation induced by the bacterium corresponded with *C. jejuni *invasion. *C. jejuni *81–176 was more invasive (*P *< 0.001) than *C. jejuni *CHR213 and *C. fetus *CHR105, as assessed by *in vitro *bacterial internalization assay (results not shown). Translocation of *C. jejuni *81–176 (*P *= 0.003) and *C. jejuni *CHR213 (*P *= 0.02) was greater than that of *C. fetus *CHR105 (Figure 5A, black bars). Translocation of *C. jejuni *81–176 and *C. jejuni *CHR213 did not differ (*P *= 0.45). All three *Campylobacter *strains, regardless of their degree of invasiveness, significantly promoted (*P *≤ 0.04) *E. coli *translocation (Figure 5A, white bars); there was no difference (*P *≥ 0.39) in the amount of *E. coli *translocation induced among the three *Campylobacter *strains. None of the *Campylobacter *strains caused changes in transepithelial electrical resistance (TER) during the 4 h experimental period (final TER was 97.7 ± 4.1%, 98.3 ± 6.8%, 94.0 ± 1.5%, and 85.6 ± 4.1% of the pre-treatment TER for control, *C. jejuni *81–176, *C. jejuni *213, and *C. fetus *CHR 105 – treated monolayers, respectively; *P *≥ 0.18).

**Figure 6:**
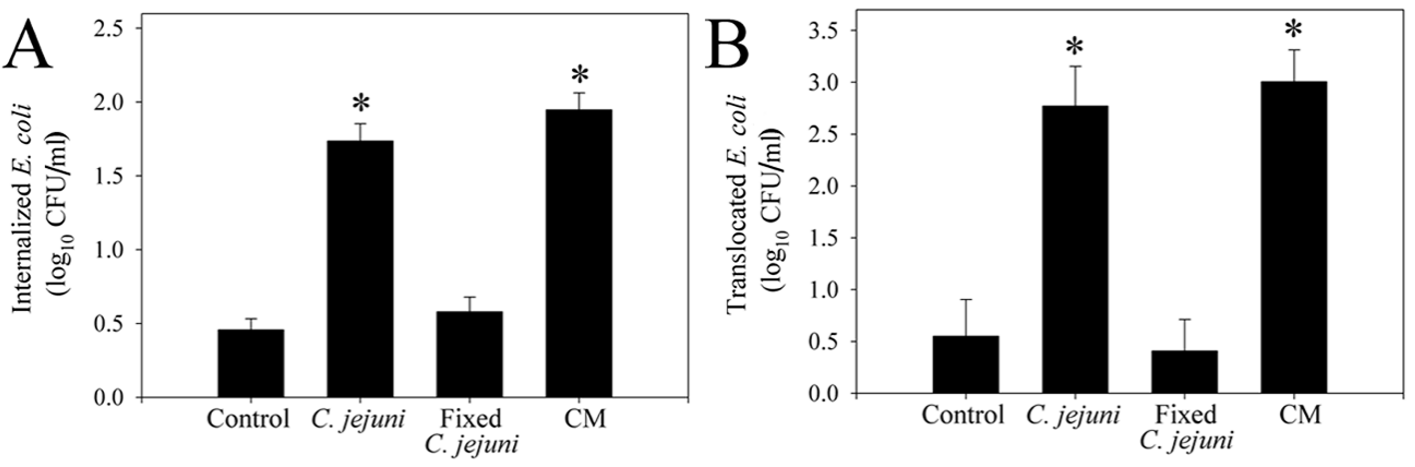
**An extracellular product from *C. jejuni *may induce *E. coli *translocation**. (A) Internalization, and (B) translocation of *E. coli *HB101 is induced by live *C. jejuni *81–176 and conditioned cell culture medium (CM), but not paraformaldehyde-fixed *C. jejuni*. Data are means ± SEM (n = 3). * *P *< 0.05 compared to controls.

Since the presence of a functional flagellum is required for *C. jejuni *invasion, studies assessed the ability of flagella defective mutants to cause *E. coli *translocation. Isogenic FlaAFlaB mutants of *C. jejuni *81–176 and CHR213 were less invasive than the wild-type (results not shown). Similarly, translocation of the isogenic FlaAFlaB mutants of *C. jejuni *81–176 and CHR213 was less (*P *≤ 0.004) than that of the wild-type (Figure 5B). Translocation of *E. coli *across the *C. jejuni*-treated monolayers did not differ (*P *≥ 0.13) between monolayers treated with the FlaAFlaB mutants or the wild-type *C. jejuni *strains (Figure 5B). The final TER did not differ between treatments (results not shown). Internalization of *E. coli *was greater in monolayers treated with live *C. jejuni *81–176 (*P *< 0.001) and conditioned cell culture media (*P *< 0.001), but not with paraformaldehyde-killed *C. jejuni *(Figure 6, *P *= 0.32). Similarly, *E. coli *translocation was greater in monolayers treated with live *C. jejuni *81–176 (*P *= 0.005) and conditioned cell culture medium (*P *= 0.002), but not paraformaldehyde-killed *C. jejuni *(*P *= 0.77).

## Discussion

Translocation of commensal bacterial antigens across the intestinal epithelium occurs at a low rate through a highly regulated process, and may play roles in establishing immunological tolerance and mucosal surveillance [18-20]. However, deregulation of this process is thought to contribute to IBD. Since campylobacteriosis is a risk factor for IBD [4,6], this study sought to determine whether *Campylobacter *induces translocation of commensal bacteria, and to characterize underlying mechanisms. The present findings indicate that *C. jejuni *increases translocation of intestinal bacteria to the MLNs, liver, and spleen of infected mice. Results from *in vitro *studies demonstrate that *C. jejuni*-induced translocation of non-invasive *E. coli *occurred in the absence of altered permeability and was mediated by lipid rafts. Moreover, wild type as well as invasion-defective *C. jejuni *mutants were able to promote translocation, but not paraformaldehyde-killed bacteria. The effect appears to involve an extracellular bacterial product that has yet to be identified. Together, the findings outline a novel mechanism through which *Campylobacter *may promote the translocation of commensal bacteria. Additional research is now needed to determine whether this mechanism may explain at least in part, how acute enteritis may contribute to inflammatory relapse in patients with IBD.

Translocation of luminal bacteria may occur via paracellular and transcellular pathways. *In vivo*, bacteria may also be transported to the MLNs and spleen by M cells or dendritic cells that sample luminal bacteria [18]. Thus, to minimize confounding effects of these cells, we used T84 colonic monolayers to model the intestinal epithelium. This also enabled assessment of the effect of invasion-defective *C. jejuni *flagella mutants that do not readily colonize the intestinal tract of animals *in vivo *[21,22].

*C. jejuni *can disrupt enterocyte tight junctions, however this effect is typically observed after prolonged incubation (>24 h) [16,17]. Present findings indicate that *C. jejuni *rapidly induces translocation of non-invasive *E. coli *across an intact intestinal epithelium via a transcellular route (i.e., <4 h post-infection). Thus, *C. jejuni *induces translocation of non-invasive *E. coli *well in advance of tight junction disruption, via a transcellular pathway. This is supported by the observed increase in the number of intracellular *E. coli *detected within *C. jejuni*-treated enterocytes using the gentamicin protection assay and confocal laser imaging. Furthermore, the barrier function of *C. jejuni*-treated monolayers remained intact (as indicated by the lack of altered permeability or TER), despite increased translocation.

While a broad range of enteropathogens, including *C. jejuni*, are known to disrupt epithelial tight junctions and increase paracellular permeability [23], very few studies have examined the effect this has on bacterial translocation. Recently, *Salmonella enterica *was shown to concurrently disrupt tight junction integrity of T84 enterocyte monolayers and promote translocation of both itself and non-pathogenic *E. coli *within 2 h post-infection [24]. Although the authors of this study did not consider the transcellular pathway in their experimental design, their results suggests that unlike *C. jejuni*, *Salmonella *induces bacterial translocation via a paracellular mechanism.

In enterocytes, transcellular translocation of commensal bacteria may occur during conditions of inflammatory and metabolic stress [10,13], suggesting that *Campylobacter *could be acting as a stressor and induce translocation via similar pathways as these stimuli. Current findings are consistent with observations that enterocytes of IBD patients display elevated transcellular antigen transport [25,26], and occasionally contain intracellular bacteria [11]. The mechanisms and signalling pathways responsible for the increased internalization and translocation of intestinal bacteria in enterocytes of IBD patiens has yet to be examined.

Lipid rafts are cholesterol and sphingolipid enriched microdomains of the plasma membrane that contain numerous receptor and signalling molecules. Bacteria can cross epithelial barriers via a transcellular route by exploiting lipid rafts [27]. Lipid raft-mediated translocation may be advantageous to translocating organisms, as bacteria endocytosed via this process appear to avoid lysosomal fusion [27]. As lipid raft-mediated endocytosis has been recently shown to mediate transcellular translocation of commensal *E. coli *in IFNγ-stimulated enterocytes [13], experiments assessed the involvement of lipid rafts in mediating *C. jejuni*-induced *E. coli *translocation. A pharmacological approach was used since confluent T84 enterocytes are notoriously resistant to transfection [28]. Disruption of lipid rafts by cholesterol depletion prevented *C. jejuni*-induced *E. coli *internalization and translocation. Conversely, increasing the plasma membrane cholesterol potentiated *C. jejuni*-induced *E. coli *internalization. In *C. jejuni*-treated monolayers, cholesterol depletion also prevented association of *E. coli *with lipid rafts, as determined by sucrose gradient fractionation. Additionally, microscopic analysis reveled that *E. coli *co-localized with the lipid raft markers, cholesterol and caveolin-1, in *C. jejuni*-infected monolayers. *C. jejuni *also appears to cause accumulation of cholesterol on the enterocyte surface, possibly indicating the coalescence of lipid rafts, making them available for exploitation by a normally non-invasive *E. coli *strain. Similarly, it has been suggested that raft coalescence may bring into close proximity, endocytic signalling molecules and bacterial binding sites, which in turn facilitate IFNγ-induced *E. coli *translocation [13]. Taken together, these observations demonstrate for the first time that *C. jejuni *may induce internalization of non-invasive intestinal bacteria via lipid raft-mediated endocytosis. Additional research is needed to further characterize the molecular mechanisms of lipid raft-mediated translocation *in vivo*. However, mice deficient in the lipid raft regulatory protein, caveolin-1, display global defects in innate immune responses and are more susceptible to infection with enteric pathogens [29,30], which may necessitate the development of mice with enterocyte-specific deletion of caveolin-1 to further advance these studies.

Invasive pathogens such as the dental pathogen, *Fusobacterium nucleatum*, can adhere to and directly transport non-invasive *Streptococcus *into oral epithelial cells [31]. It was recently observed that *C. jejuni *itself may translocate enterocytes via lipid rafts [32] or via clathrin-mediated endocytosis [33,34]. However, the present findings that highly invasive (strain 81–176), moderately invasive (CHR213), and modestly invasive campylobacter's (CHR105 and FlaAFlaB mutants) caused equivalent *E. coli *translocation, suggest that *Campylobacter *does not directly 'shuttle' non-invasive bacteria into enterocytes. Furthermore, conditioned media also induced *E. coli *internalization and translocation, indicating that a hitherto unidentified extracellular product may be responsible for the observed effect.

## Conclusion

Findings that *Campylobacter *disrupts intestinal epithelial transcellular transport and promotes translocation of non-invasive bacteria may have important implications in mucosal inflammatory responses towards the intestinal microflora. Future studies will assess whether and how this inflammatory response may result in collateral damage, or could exacerbate or initiate IBD in susceptible individuals. *Campylobacter*-infected enterocytes offer a unique model for further investigation into mechanisms promoting commensal translocation, and may improve our understanding of the pathogenesis of inflammatory bowel diseases.

## Methods

### Bacteria and culture conditions

*C. jejuni *81–176, a reference clinical strain [35], was used throughout this study. *C. jejuni *CHR213 and *C. fetus *CHR105 are clinical isolates that were characterized as previously described [36]. FlaAFlaB mutants of *C. jejuni *81–176 and CHR213 were constructed and characterized as described previously [36]. Intestinal colonization of mice was reduced for the *C. jejuni *81–176 FlaAFlaB mutant (results not shown), confirming previous observations [21,22].

Inoculum was prepared by growing *Campylobacter *for 14–16 h in CYE broth (37°C, 100 rpm, microaerobic atmosphere) [37]. *E. coli *HB101 was grown for 14–16 h in Columbia broth (37°C, 100 rpm; Difco, Detroit, MI).

### Fluorescent labelling of *E. coli*

*E. coli *were washed with NaHCO_3 _buffer (0.1 M, pH 8.3), and incubated for 45 min in Alexa-488 carboxylic acid succinimidyl ester (0.5 mg/ml NaHCO_3 _buffer; Molecular Probes, Eugene, OR). *E. coli *were then washed and suspended (≈10^9 ^CFU/ml) in PBS containing 2% NaHCO_3 _(mouse study) or antibiotic-free Dulbecco's modified Eagle medium (DMEM)/F-12 (T84 studies). Labelling did not affect bacterial viability (results not shown).

### *In vivo *study

Mice (Balb/c; Charles River, Montreal, QC) were housed at Agriculture and Agri-Food Canada, Lethbridge (Alberta) under the guidelines established by the Canadian Council on Animal Care. Procedures were approved by institutional Animal Care and Biosafety Committee's. Five-week-old female mice were inoculated with *C. jejuni *81–176 (10^8 ^CFU in CYE containing 2% NaHCO_3_) or sterile CYE (controls) by gavage on days one and two. To examine translocation of a model commensal intestinal bacterium, all mice were inoculated on day three with fluorescent non-invasive *E. coli *(10^8 ^CFU) transformed with an ampicillin resistant (Amp^R^) plasmid (pGEM-T Easy, Promega, Madison, WI). Four hours later, mice were euthanized and the MLNs, and sections of liver, spleen, and ileum were aseptically removed. Fecal pellets were also collected prior to euthanasia. Tissues and feces were weighed and homogenized in PBS. *C. jejuni*, microaerobic bacteria, and *E. coli *were enumerated by spreading serial dilutions of fecal or tissue homogenates onto Karmali agar containing selective supplement SR167 (Oxoid, Nepean, ON), non-selective Karmali agar, and MacConkey agar containing ampicillin (100 μg/ml; Difco), respectively, and incubating media at 37°C in a microaerobic (*C. jejuni*, microaerobic bacteria) or ambient (*E. coli*) atmosphere. Isolated bacteria were identified by PCR and comparative 16S rRNA sequence analysis.

### Epithelial cell culture

T84 human colonic epithelial cells (passages 5 to 15; American Type Culture Collection, Manassas, VA) were grown in DMEM/F-12 plus 10% foetal bovine serum, 200 mM L-glutamine, 100 U/ml penicillin, 100 μg/ml streptomycin, 80 μg/ml tylosin, and incubated at 37°C and 5% CO_2_. Medium was replenished every 2 to 3 days. For translocation studies, cells were seeded onto Transwell filters at 4 × 10^5 ^cells/filter (5 μm pore size, 1.13 cm^2^; Costar, Corning Inc. Corning, NY). Transepithelial electrical resistance (TER) was monitored with an electrovoltohmeter (World Precision Instruments, Sarasota, FL), and monolayers were used at confluence (TER > 1000 Ω/cm^2^). For microscopy, cells were seeded into Lab-Tek chamber slides at 8 × 10^4 ^cells/well (Nalgene Nunc International, Naperville, IL).

### *In vitro *bacterial translocation and internalization assay

Monolayers were washed with PBS and antibiotic-free DMEM/F12 was added to the apical and basal compartments. Monolayers were inoculated apically with *E. coli *± *Campylobacter *or to achieve a MOI of 100 CFU/enterocyte for each bacterial species. Control monolayers received an equivalent volume of CYE. Following 4 h incubation in microaerobic conditions, *E. coli *and *Campylobacter *recovered in the basal compartment medium were enumerated by spreading serial dilutions onto non-selective Karmali agar and incubating microaerobically at 37°C. These conditions are suitable for the growth of *E. coli *and *Campylobacter*. T84 cells maintain TER > 1000 Ω/cm^2 ^for > 24 in microaerobic atmosphere (data not shown).

To assess *E. coli *internalization, monolayers washed with PBS and incubated for 1 h with DMEM/F12 containing gentamicin (250 μg/ml; Sigma-Aldrich, Oakville, ON) [10]. Monolayers were washed, lysed with 0.1% Triton X-100/PBS, and viable bacteria were enumerated. A preliminary experiment confirmed that *E. coli *were killed by the gentamicin treatment. *Campylobacter *internalization was determined as for *E. coli*, except monolayers were incubated for 3 h with DMEM/F12 containing gentamicin (500 μg/ml), as described previously [36].

For some experiments, apical compartments were inoculated with *E. coli *± paraformaldehyde-fixed *C. jejuni *81–176 or apical medium was replaced with conditioned DMEM/F12. Paraformaldehyde-fixed *C. jejuni *were prepared by incubating bacteria for 2 h in 2% paraformaldehyde. Cells were washed and suspended in antibiotic-free DMEM/F12 (≈10^9 ^CFU/ml). To prepare conditioned medium, T84 monolayers (75 cm^2 ^flask) were washed with PBS, and antibiotic-free DMEM/F12 was added to the flask. Cells were inoculated with *C. jejuni *81–176 (MOI 100) and incubated microaerobically overnight (37°C). Media was clarified by centrifugation, followed by filtration through a 0.2 μM syringe filter. Sterility was confirmed by viable counting.

### Epithelial permeability

Following the infection period, monolayers were washed with sterile Ringer's solution. A 3 kDa FITC-dextran probe (500 μl, 100 mM in Ringer's solution; Molecular Probes) was added to the apical compartment, and 1 ml of Ringer's solution added to the basal compartment and incubated for 3 h at 37°C, as describe previously [38]. Samples were collected from the basal compartment and absorbance_485 _was measured. Data were expressed as % apical dextran/cm^2^/h.

### Manipulation of plasma membrane cholesterol

Disruption of lipid rafts was performed by membrane cholesterol depletion as validated previously. Monolayers received MβCD (50 mM or 10 mM; Sigma) plus lovastatin (1 μM; Sigma) 30 min prior to inoculation. Conversely, plasma membrane cholesterol was increased by incubating monolayers for 4 h with soluble βCD-complexed cholesterol (100 μM; Sigma) and washing with PBS prior to inoculation. These treatments did not affect viability of T84 cells, *C. jejuni*, or *E. coli *(results not shown). Plasma membrane cholesterol was measured using an Amplex red cholesterol assay kit (Molecular Probes). Total protein was measured using a Bradford protein assay (Bio-Rad Laboratories, Hercules, CA).

### Microscopy

T84 apical membranes were fluorescently labelled by washing monolayers with Hank's buffered saline (HBSS) and incubating with Alexa-546 carboxylic acid succinimidyl ester (0.5 mg/ml HBSS, 1 h, 4°C, Molecular Probes). Monolayers were washed and inoculated with Alexa488-labelled *E. coli *± *C. jejuni*, as described for the translocation assay. After incubation, slides were washed with PBS, and fixed in paraformaldehyde (2%). Optical sectioning was carried out by confocal laser microscopy (Leica Microsystems GmbH, Wetzler, Germany). Digitized images were analyzed with Imaris software (Bitplane AG, Zurich, Switzerland).

To visualize cholesterol-rich domains, T84 cells were stained with the fluorescent sterol-binding drug, filipin (0.5 mM in PBS; Sigma), for 30 min [39]. To stain for caveolin-1, slides were rinsed with PBS, incubated with glycine (1% in PBS) for 15 min, and rinsed with PBS. Cells were permeabilized for 10 min with Triton X-100 (0.5% in PBS), blocked with BSA (2% in PBS), incubated with mouse anti-caveolin-1 antibodies (1/100 dilution in PBS; BD Bioscience, San Diego, CA) followed by Alexa-488 conjugated anti-mouse IgG (1/100 dilution in PBS; Molecular Probes).

### Lipid raft isolation

Another set of experiments characterized the association of translocating *E. coli *with lipid rafts. Monolayers were inoculated with fluorescent *E. coli *± *C. jejuni *as described for the translocation assay. Lipid rafts were isolated by sucrose gradient fractionation, as previously described [39,40]. For each of eight fractions that were collected, fluorescent *E. coli *were quantified by measuring absorbance_485_, and the lipid raft marker, caveolin-1, was detected by western blot analysis using mouse anti-caveolin-1 antibodies (1/1000 dilution; BD Bioscience), and HRP-conjugated anti-mouse IgG antibodies (1/5000 dilution; Sigma) [39].

### Statistical analysis

Experiments were conducted ≥ three times independently. Assays were conducted at least in triplicate, and mean values were used for analysis. Analyses were performed with GraphPad InStat software (GraphPad Software Inc., San Diego, CA). Data are expressed as means ± SEM. Data with ≥ three treatments were compared by one way ANOVA, followed by the protected Tukey-Kramer multiple comparison test. Data with two treatments were compared using an unpaired Student's *t*-test. Translocation incidences in mice were compared using the Fisher's exact test. *P *≤ 0.05 was considered significant.

## Authors' contributions

LKT participated in designing and conducting experiments, and writing the manuscript. GDI participated in experimental design, statistical analysis, and editing the manuscript. AGB participated in experimental design and writing the manuscript. All authors read and approved the final manuscript.
